# Unexpected Effect of Digestion Products of Infant Formula in Enhancing the Solubilisation of Tolfenamic Acid During Digestion

**DOI:** 10.3390/pharmaceutics18040480

**Published:** 2026-04-14

**Authors:** Thomas Eason, Malinda Salim, Vanessa Zann, Ben J. Boyd

**Affiliations:** 1 Drug Delivery Disposition and Dynamics, Monash Institute of Pharmaceutical Sciences, Monash University (Parkville Campus), 381 Royal Parade, Parkville, VIC 3052, Australia; thomas.eason@monash.edu (T.E.);; 2 Quotient Sciences, Nottingham NG11 6JS, UK; 3 Department of Pharmacy, University of Copenhagen, Universitetsparken 2, 2100 Copenhagen, Denmark

**Keywords:** lipid based formulation, in vitro lipolysis, drug solubilisation, infant formula, tolfenamic acid

## Abstract

**Background/Objectives**: Recent studies have shown that the solubilisation of poorly water-soluble drugs can be enhanced by using infant formula as a lipid-based formulation. In those studies, digestion of the triglycerides in infant formula to produce more polar lipids, namely fatty acids and monoglycerides, produced a high-capacity solubilisation environment for weakly basic drugs such as clofazimine, driven mainly by ion-pairing of the fatty acid with the drug. However, digestion of lipid-based formulations is not expected to provide the same effect for nonionised or acidic drugs and in fact may present a reduced solubilisation capacity for weakly acidic drugs. **Methods:** In this study, a weakly acidic drug, tolfenamic acid, was dispersed in reconstituted infant formula, and the infant formula was digested under in vitro simulated intestinal conditions. The quantity of tolfenamic acid that was solubilised in the infant formula during digestion was determined by high-performance liquid chromatography and small-angle X-ray scattering. **Results:** Unexpectedly, digestion of the infant formula increased the solubilisation capacity for tolfenamic acid. Reconstituting infant formula at a higher fat content also increased the rate and extent of solubilisation of tolfenamic acid during digestion. The quantity of tolfenamic acid that was solubilised during digestion correlated approximately linearly with the quantity of free fatty acids produced during digestion. **Conclusions:** These results show that a weakly acidic drug can also exhibit digestion-driven solubilisation in a lipid-based formulation in the absence of ion-pairing and highlights the need to better understand drug response to digestion of lipid-based foods and formulations, and their versatility as a formulation option even for poorly water-soluble acidic drugs.

## 1. Introduction

Lipid-based formulations (LBF) can be used to raise the apparent solubility of poorly water-soluble drugs in the gastrointestinal tract and thereby enhance their absorption [1]. During digestion, glyceride-based lipids such as triglycerides or partially digested mono-, di- and triglyceride commercial mixtures used in the formulations are broken down into free fatty acids and monoglycerides which can self-assemble with endogenous surfactants in the small intestine to form colloidal liquid crystal structures including mixed micelles and vesicles [2,3,4]. The solubilisation capacity of those colloidal structures is a critical determinant of enabling drug to be in a dissolved state available for absorption. If the process of digestion yields a less-favourable environment for drug to remain dissolved then precipitation is likely to occur with resulting limited bioavailability.

In this context, milk has received increasing attention as a paediatric-friendly LBF [5,6,7]. Milk contains emulsified fats (98% triglycerides, TAG) which enable it to solubilise lipophilic, poorly water-soluble drugs [7,8]. It was demonstrated that even drugs with limited solubility in undigested milk, when prepared as a suspension, can be dissolved during the digestion process, opening the possibility of the ‘chaser’ approach to co-formulate and/or co-administer the lipid formulation with solid drug substance [8,9]. In terms of possible dose forms, for co-formulation it may involve mixing drug powder with infant formula in a sachet or formulating it into a dispersible tablet, while for co-administration, a tablet could be consumed with a known mass of reconstituted powder or even an infant formula filled capsule if it contained a sufficient amount of fat. While milk can improve the oral bioavailability of poorly water-soluble drugs, it is unlikely to be approved as an excipient because it is a biological product with inherently variable composition. An alternative is infant formula, which is a standardised nutritional matrix and already has a tightly regulated composition. Infant formula contains a mixture of bovine-derived and vegetable-derived triglycerides. Digested infant formula has been shown to have a superior solubilisation capacity for poorly water-soluble drugs to bovine milk [10,11], although their in vivo efficacy has only recently been demonstrated [12].

While milk and infant formula have been shown to solubilise a variety of drugs during digestion, the majority of those drugs were weakly basic [8,10,11,13]. It has previously been reported that digesting model LBF increased their solubilisation capacity for weakly basic drugs, whilst it decreased their solubilisation capacity for weakly acidic and non-ionisable drugs [14]. The enhanced solubilisation of weakly basic drugs in digested LBF was attributed to electrostatic interactions between positively-charged drug molecules and oppositely-charged free fatty acids, which attracted drug molecules into fatty acid-rich mixed micelles and assisted their solubilisation, however this mechanism is not available for weakly acidic drugs.

**Figure 1:**
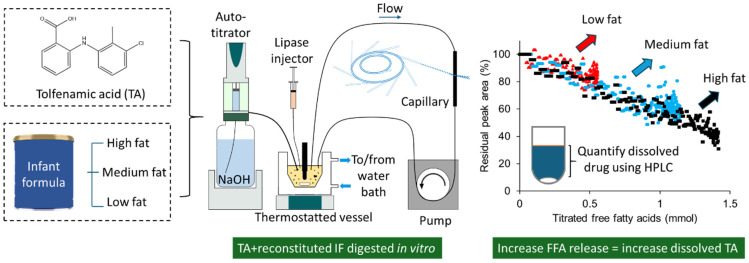
Overview of the study and its findings. Tolfenamic acid (TA) was suspended in reconstituted infant formula and digested using an in vitro lipolysis model. The dissolution of TA in reconstituted infant formula before and during lipolysis was tracked using small-angle X-ray scattering. HPLC was used to determine the solubility of TA in digested and undigested infant formula.

The aim of this study was therefore to investigate whether weakly acidic drugs exhibit digestion-driven drug solubilisation in infant formula, and whether the production of fatty acids during the digestion of infant formula played a significant role in the solubilisation of weakly acidic drugs. Tolfenamic acid (TA), a model poorly water-soluble weakly acidic drug (aqueous solubility < 20 μg/mL, log P 5.49, pKa 5.11) was selected because it is a well-studied compound that is “rich” in polymorphs [15] and its solubility in (non-milk-based) LBF has been previously reported [16,17]. Tolfenamic acid was suspended in reconstituted infant formula and the lipids digested under in vitro simulated intestinal conditions (Figure 1). Small-angle X-ray scattering (SAXS) was used to monitor the solubilisation of tolfenamic acid in situ during digestion, while high-performance liquid chromatography (HPLC) was used to quantify solubilised tolfenamic acid in aliquots collected during digestion.

## 2. Materials and Methods

### 2.1. Materials

Blackmores Stage 1 infant formula™ was purchased from local suppliers (Chemist Warehouse, Brunswick, VIC, Australia). Table S1 in the Supplementary Materials lists the compositional information of the infant formula. Tolfenamic acid (>99% purity) was purchased from AK Scientific (Union City, CA, USA). Trizma^®^ maleate (reagent grade), sodium azide (≥99%) and calcium chloride dihydrate (>99% purity) were purchased from Sigma-Aldrich (St. Louis, MO, USA). Sodium chloride (>99% purity) and sodium dihydrogen phosphate (>99% purity) were purchased from Chem-Supply (Gillman, SA, Australia). Sodium hydroxide pellets (>97% purity) and orthophosphoric acid (85% *w*/*v*) were purchased from Ajax Finechem (Seven Hills, NSW, Australia). Hydrochloric acid (32% *w*/*v*) was purchased from RCI Labscan (Pathum Wan, Bangkok, Thailand). FaSSIF/FeSSIF/FaSSGF powder was purchased from Biorelevant.com Ltd. (London, UK). USP grade pancreatin extract was purchased from Southern Biologicals (Alphington, VIC, Australia). Orlistat (>97.5% purity) was purchased from Selleck Chemicals (Houston, TX, USA). Acetonitrile (chromatography grade) and methanol (chromatography grade) were purchased from Merck (Darmstadt, Germany). All chemicals were used without further purification. Water was sourced from a Millipore Milli-Q purification system (Merck Millipore, Burlington, MA, USA).

### 2.2. In Vitro Digestion of Reconstituted Infant Formula Containing Tolfenamic Acid

In vitro digestion experiments were conducted via previously described methods [18]. Briefly, tolfenamic acid (TA, 25 mg) was mixed with fed-state simulated intestinal fluid (5 mL, containing 15 mM sodium taurocholate and 3.75 mM lecithin) to produce a suspension. The suspension was added to a thermostatted glass vessel (37 °C) along with 18 mL of reconstituted infant formula. The contents of the vessel were magnetically stirred (750 RPM) and the pH of the contents was adjusted to 6.500 ± 0.003. The contents of the vessel were then left to stir for a 15 min dispersion period.

After the dispersion period, a porcine pancreatin suspension (2 mL, activity of ~700 tributyrin units) was added to the vessel to digest the fat content. The pH of the digest was maintained at 6.50 by a pH-stat apparatus (Titrando 902, Metrohm AG, Herisau, Switzerland) that titrated 2 M NaOH solution into the digest. Each sample was digested for 60 min.

Infant formula was reconstituted to three different fat contents (providing 13.7, 27.4 and 41.0 mg of fat per mg of tolfenamic acid in the digest, equal to reconstituted fat contents of 1.9, 3.8 and 5.7% *w*/*v* respectively) to examine how the fat content influenced the solubilisation of tolfenamic acid. Infant formula was reconstituted in a digestion buffer (pH 6.50) containing 50 mM Tris-maleate, 150 mM NaCl, 5 mM CaCl_2_·2H_2_O and 6 mM NaN_3_.

### 2.3. Quantifying Dissolved Tolfenamic Acid in Samples Retrieved During the Digestion of Reconstituted Infant Formula Using HPLC

Samples (200 μL) were collected from the vessel at 15 min intervals during the dispersion and digestion periods. Orlistat solution (2 μL, 2 mg/mL in methanol) was added to digested samples to inhibit further digestion. All samples were ultracentrifuged at 434,900× *g* for 10 min at 37 °C in an Optima MAX-TL ultracentrifuge (Beckman Coulter, Indianapolis, IN, USA). Following ultracentrifugation, the aqueous supernatant and upper lipid layer (containing solubilised tolfenamic acid) were collected and combined. The sedimented pellet (containing undissolved tolfenamic acid) was also collected. Methanol (1 mL, containing diclofenac as an internal standard) was added to the combined aqueous/lipid phases and to the pellet. The samples were then diluted in mobile phase consisting of 60% B (methanol) and 40% A (10 mM NaH_2_PO_4_ in water, pH 2.3). The quantity of tolfenamic acid in each sample was determined by HPLC-UV. The HPLC system consisted of a Shimadzu CBM-20A system controller, LC-20AD solvent delivery module, SIL-20A autosampler, and a CTO-20A column oven set at 25 °C, coupled to an SPD-20A UV-detector (Shimadzu Corporation, Kyoto, Japan). A 6 min isocratic method (40% A and 60% B) with a reverse-phase C18 column (4.6 × 75 mm, 3.5 μm; Waters Symmetry, Milford, MA, USA) was used for each separation. Diclofenac was retained for 2.6 min while tolfenamic acid was retained for 4.5 min. The drugs were detected at 277 nm (diclofenac) and 343 nm (tolfenamic acid).

### 2.4. Tracking the Dissolution of Tolfenamic Acid During the Digestion of Reconstituted Infant Formula Using Small-Angle X-Ray Scattering

Small-angle X-ray scattering (SAXS) was used to monitor the solubilisation of tolfenamic acid in reconstituted infant formula during dispersion and digestion, using previously established methods. The in vitro digestion set-up described in Section 2.2 was coupled to the SAXS/WAXS beamline at the Australian Synchrotron (Clayton, VIC, Australia) [19]. The contents of the digestion vessel were circulated from the vessel through a peristaltic pump to a quartz capillary mounted in the X-ray beam (13 keV, wavelength λ = 0.954 Å) and back to the vessel at approximately 10 mL/min. The sample-to-detector distance was approximately 0.6 m, providing a *q*-range of 0.04 to 2.00 Å^−1^. 2D SAXS patterns from the sample were recorded during dispersion and digestion using a Pilatus 1 M detector, with a 5 s acquisition period and 1 acquisition every 20 s. The raw data were reduced to I(*q*) vs. *q* by radial integration using the in-house software ScatterBrain version 2.71. The scattering vector *q* is defined by *q* = (4π/λ)sin(2θ/2) where λ is the X-ray wavelength and 2θ is the scattering angle. The area under a characteristic diffraction peak for tolfenamic acid at *q* = 0.83 Å^−1^ was integrated and plotted as a function of time using Origin 2023b software v10.0.5.157 (OriginLab Corporation, Northampton, MA, USA).

### 2.5. Measuring the Equilibrium Solubility of Tolfenamic Acid in Reconstituted Infant Formula

The solubility of tolfenamic acid was measured in undigested and digested reconstituted infant formula. Digested reconstituted infant formula was prepared by digestion as described in Section 2.2 without drug present. The media was digested for an hour and then inhibited by the addition of orlistat solution at the same ratio described in Section 2.3. Undigested reconstituted infant formula was prepared in a similar manner, but tris buffer was substituted for pancreatin solution.

Excess crystalline tolfenamic acid was added to 6 mL of each media in triplicate and incubated at 37 °C with constant mixing. Samples (200 µL) were collected at *t* = 16, 24, 40 and 48 h. Collected samples were processed as per Section 2.3 to determine the amount of solubilised tolfenamic acid in each.

### 2.6. Statistical Analysis

Solubility data are presented as mean ± standard deviation (SD) from three independent replicates. Statistical analyses were performed using GraphPad Prism version 10 (GraphPad Software, San Diego, CA, USA). An unpaired two-tailed *t*-test with Welch’s correction was applied to assess differences between groups, with statistical significance defined as *p* < 0.05.

## 3. Results

### 3.1. Kinetics of Lipid Digestion in Reconstituted Infant Formula

**Figure 2:**
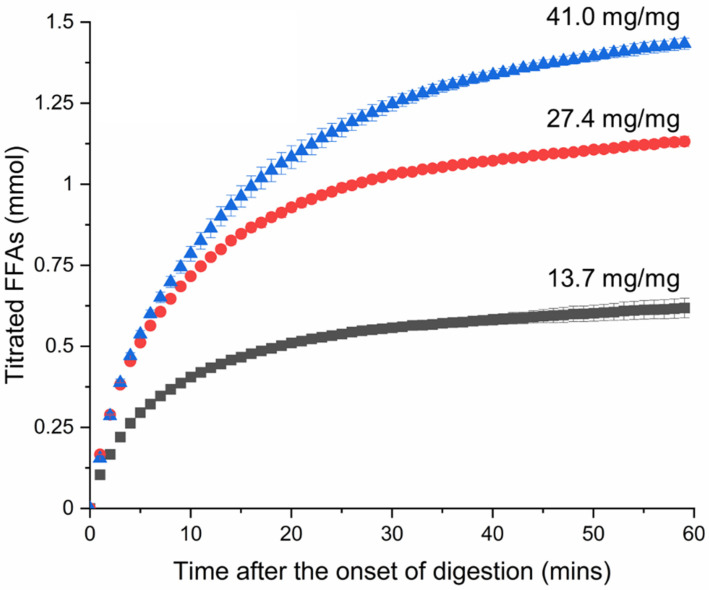
Free fatty acids (FFA) titrated during the in vitro lipolysis of reconstituted infant formula containing tolfenamic acid. Infant formula was reconstituted at three fat contents to provide 13.7, 27.4 and 41.0 mg of fat per mg of tolfenamic acid (black, red and blue respectively). The titrated FFA values are expressed as the mean ± SD (*n* = 3).

The triglyceride content in reconstituted infant formula was digested to form free fatty acids and monoglycerides during in vitro digestion. The free fatty acids were titrated to quantify the rate and extent of fat digestion. Figure 2 shows that reconstituted infant formula was digested rapidly, with over 50% of total fatty acid production occurring in the first 10 min of digestion. Raising the fat content increased the total amount of fatty acids produced during digestion. Increasing the fat/drug ratio from 13.7 to 27.4 mg/mg caused a proportional increase in the production of free fatty acids, with approximately twice as many free fatty acids being titrated over 60 min of digestion. Increasing the fat/drug ratio further to 41.0 mg/mg caused a smaller increase in total fatty acid production likely due to lipase no longer being in excess. It is worth noting that only ionised fatty acids are quantified by titration, while a fraction remains unionised and can only be determined by back-titration. For infant formula, around 50% of fatty acids are generally ionised at pH 6.5, although this varies with chain lengths of the fatty acids.

### 3.2. Solubility and Dissolution of Tolfenamic Acid in Digested and Undigested Reconstituted Infant Formula

**Figure 3:**
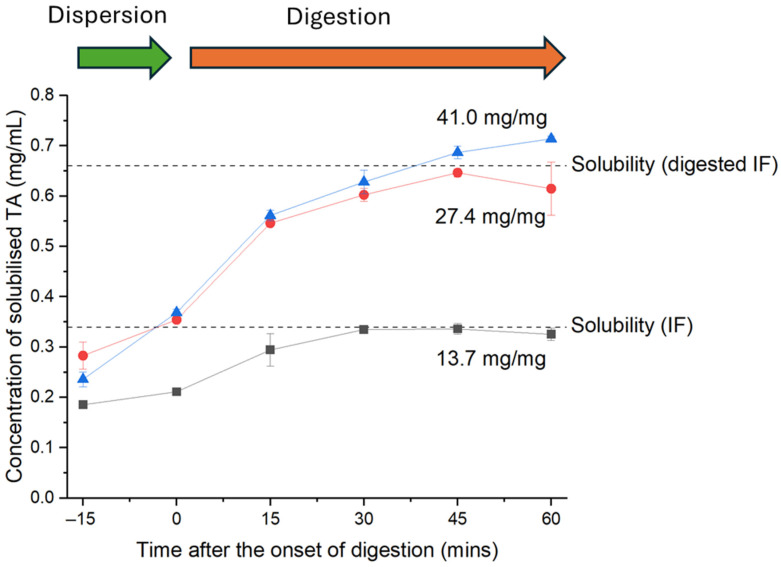
Dissolution of tolfenamic acid (TA) in reconstituted infant formula before (dispersion) and after the onset of lipid digestion, quantified using HPLC. Infant formula was reconstituted at three fat contents to produce fat/drug ratios of 13.7, 27.4 and 41.0 mg of fat per mg of tolfenamic acid. Data are mean ± SD (*n* = 3). The equilibrium solubility of tolfenamic acid in infant formula and digested infant formula (at 27.4 mg/mg) is shown as the dashed lines. Lines joining data points are to guide the eye only.

*Undigested reconstituted infant formula*: The equilibrium solubility of tolfenamic acid in undigested infant formula reconstituted to 3.8% fat (27.4 mg fat/mg of drug) was 0.34 ± 0.02 mg/mL. When crystalline tolfenamic acid was dispersed in reconstituted infant formula for 15 min prior to commencing lipolysis (the ‘dispersion phase’ *t* = −15 min to *t* = 0 min in Figure 3), the drug dissolved rapidly in digesting infant formula. Raising the fat/drug ratio from 13.7 to 27.4 mg/mg increased the quantity of tolfenamic acid that dissolved during the dispersion phase (from 19.4 ± 0.7% to 32.6 ± 0.05% of the dose). This difference was statistically significant, as determined by an unpaired two-tailed *t*-test with Welch’s correction (*p* < 0.001). In contrast, increasing the fat/drug ratio from 27.4 to 41.0 mg/mg did not significantly change the dissolution of tolfenamic acid during the dispersion phase. Notably, the amount of tolfenamic acid dissolved in infant formula after 15 min of dispersion was comparable to the equilibrium solubility of tolfenamic acid in undigested infant formula.

As seen in Figure 3, the dashed line indicates the solubility of tolfenamic acid in infant formula and reflects the maximum amount of drug that could be dissolved at equilibrium in digested infant formula, which is supported by the dynamic solubilisation that appears to plateau at that fat-to-drug ratio.

*Impact of digestion on solubility and dissolution of tolfenamic acid*: The solubility of tolfenamic acid in digested infant formula (0.66 ± 0.1 mg/mL, upper dashed line in Figure 3) was significantly greater than in undigested reconstituted infant formula (lower dashed line), as was determined by an unpaired two-tailed *t*-test with Welch’s correction (*p* < 0.05). Figure 3 shows that approximately 50% of the tolfenamic acid that dissolved during the digestion phase (0 min to +60 min) did so in the first 15 min after commencing digestion and approached the measured solubility of drug in digested infant formula (indicated by the upper dashed line).

**Figure 4:**
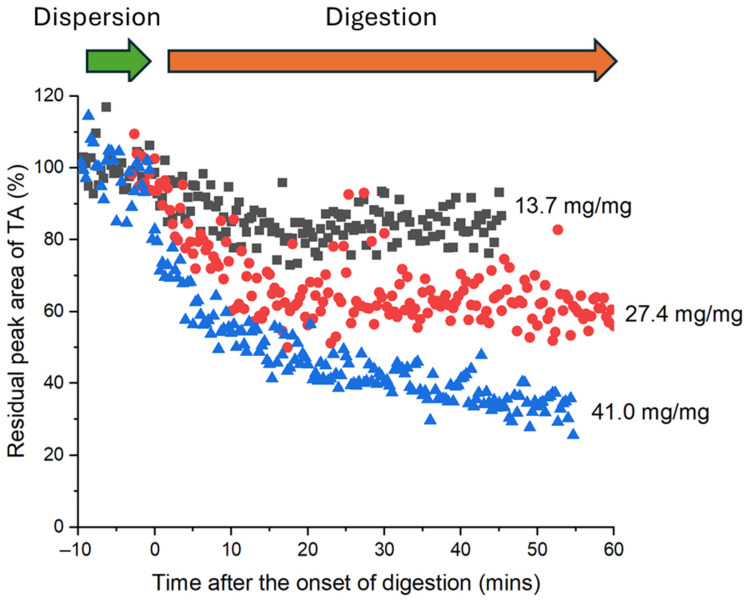
Residual quantity of crystalline tolfenamic acid (TA) suspended in reconstituted infant formula during dispersion and in vitro digestion at three fat/drug ratios determined from the area of the characteristic Bragg peak at *q* = 0.83 Å^−1^.

The trend in kinetics and extent of solubilisation from HPLC measurements was confirmed using in situ small-angle X-ray scattering during digestion (Figure 4). The peak area of crystalline tolfenamic acid at *q* = 0.83 Å^−1^ (a major Bragg peak characteristic to tolfenamic acid; Figure S1) did not change substantially during the initial dispersion period (consistent with the limited dissolution in the dispersion phase measured using HPLC in Figure 3). However, during digestion there was clear reduction in the residual peak area, indicating that during the digestion phase drug was solubilised more effectively by the evolving lipid compositions changing from triglycerides to monoglycerides and fatty acids. Note that all digestions in Figure 4 commenced with the same concentration of drug but different amounts of fat. Increasing the drug/fat ratio caused an increased proportion of tolfenamic acid to dissolve during the digestion phase. The amount of tolfenamic acid dissolved after 60 min of lipolysis increased from 32.6% to 61.5% when the fat/drug ratio was increased from 13.7 to 27.4 mg/mg. Increasing the drug/fat ratio further to 41.0 mg/mg caused a smaller increase in the total amount of tolfenamic acid that dissolved during digestion.

Importantly, no new Bragg peaks emerged in the diffraction profiles throughout the digestion process, precluding the changes in peak area being due to a polymorphic transformation (Figure S2). The polymorphic form of tolfenamic acid used in this study is form II.

**Figure 5:**
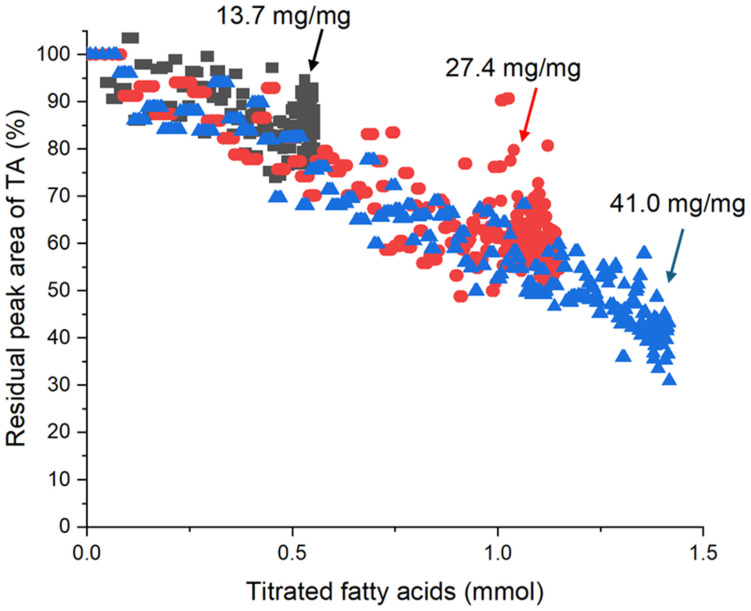
Residual crystalline tolfenamic acid (TA) remaining in reconstituted infant formula during in vitro digestion (as determined by the area of its characteristic Bragg peak at *q* = 0.83 Å^−1^) as a function of the quantity of free fatty acids produced during lipolysis.

To further analyse the impact of digestion and the relationship between the generation of fatty acids through digestion of triglyceride and drug solubilisation, the residual peak area was replotted as a function of fatty acid produced rather than time (Figure 5). It is clear from Figure 5 that there is an almost strictly linear dependence between the fatty acids present and drug solubilisation, independent of the starting amount of triglyceride. This is an extremely strong indicator that generation of free fatty acids is driving increased solubilisation of tolfenamic acid, even though tolfenamic acid is itself an acidic drug, a major point of discussion below.

## 4. Discussion

Dilution and digestion of oral lipid-based formulations in the gastrointestinal tract can have a major impact on their performance. LBFs can be prepared with a wide range of compositions from high surfactant and solvents content with little or no digestible lipids in the formulation (so-called ‘Type 4’ formulations) through to those containing only triglycerides (Type 1 formulations) [20]. Generally, development of LBFs has focussed on drug being in solution in the LBF as a formulation, then attempt to tune composition to retain the drug in solution after administration, or to generate a consistent supersaturation effect to drive absorption. For Type 4 formulations dilution is the primary concern; these formulations are considered for the maximal dose of drug dissolved in the formulation but carry a high risk as surfactants and solvent separate from the drug and although a potentially favourable supersaturated condition may exist for a period of time, there is a high chance of drug precipitation. In contrast, Type 1 formulations contain only oil and rely entirely on digestion and dispersion through lipolysis and generation of polar lipids from the triglycerides. Drug is often less soluble in triglycerides than in more polar lipids or surfactants and are not dispersible into an emulsion or nano-sized emulsion as the other three types, hence Type 1 formulations are less well regarded in a formulation development context even though they may offer superior bioavailability [21].

Strategies to prevent digestion-driven precipitation of drugs from LBFs are therefore of interest. One strategy is to add polymeric precipitation inhibitors to the formulation to maintain the drug in a supersaturated state during digestion [22,23]. Other research has focussed on redirecting drug precipitation from crystalline forms to amorphous forms with better dissolution characteristics. For example, the weakly basic drug cinnarizine was shown to precipitate from a LBF during digestion as an amorphous ion pair with oleic acid [24]. The ion-pair redissolved in gastrointestinal fluids much more rapidly than crystalline cinnarizine, improving its chances of absorption. Similarly, the weakly acidic drug tolfenamic acid has been shown to precipitate from a LBF as an amorphous ion-pair with the cationic lipid didodecyl ammonium bromide (DDAB) [17]. This approach to controlling precipitation is made more complex for weakly acidic drugs as the lipophilic cationic counter-ions are typically cytotoxic. It is possible to overcome this problem with synthetic, biocompatible counter-ions but such solutions are complex. In all cases above, the excipients in the LBFs are also not able to be used in infant or paediatric formulations, limiting application to those patient groups on toxicity grounds.

Milk (in powder form) and infant formula could be considered as Type 2 formulations, dispersing readily when reconstituted to form emulsions with a range of particle sizes but without the need for synthetic surfactants for solvents [7]. Upon dilution the fat droplets retain drug in a dissolved form, and it is not until the lipids are digested that the fate of co-formulated or co-administered drug is largely determined, either providing a more favourable environment into which drug will remain in solution or dissolve if present in solid form, or if the environment is less favoured drug may precipitate, or dissolve to an insufficient extent to solubilise a full dose of drug. For both milk and infant formula, most past research has focussed on drug being pre-dissolved in the emulsion under the typical lipid-based formulation paradigm. However, recent efforts to consider co-formulation as a suspension of drug particles in milk and infant formula, or co-administration of lipids with a solid dose form have shown promise both in in vitro dissolution testing during digestion [11,18,25,26,27], and in in vivo bioavailability studies [12,26,28].

One approach to anticipating the impact of digestion on the performance of a LBF is to select a LBF based on the difference in drug solubility before and after digestion, where a significantly greater solubility in the post-digestion colloidal media indicates a strong sink for dissolution of the drug. This is particularly relevant for weakly basic drugs, which can have enhanced solubility in colloidal lipid digestion products (such as mixed micelles) courtesy of ion-pairing interactions with free fatty acids. This was demonstrated in a study by Alskar et al., where the weakly basic drugs haloperidol and cinnarizine had markedly higher solubility in pre-digested LBF than in the parent LBF [14]. Neutral and weakly acidic drugs in that study could not ion-pair with free fatty acids produced during digestion and thus had lower solubility in the digested LBF than in the parent LBF. This however contrasts with the current study, where the solubility of weakly acidic tolfenamic acid in the pre-digested infant formula was at least twice that in undigested reconstituted infant formula at the same fat content (Figure 3). Consistent with these results, tolfenamic acid was previously reported to have 3.7-fold greater solubility in long-chain triglyceride digestion products (Maisine 35-1) than in the parent long-chain triglycerides (soybean oil) [29]. We therefore would anticipate from solubility measurements that tolfenamic acid would exhibit digestion-driven solubilisation in a LBF rich in long-chain triglycerides as is the case for infant formula which generally is constituted with vegetable oils high in oleic acid.

Accordingly, the production of free fatty acids and monoglyceride during the digestion of infant formula had a direct impact on the dissolution of tolfenamic acid. SAXS data (Figure 5) showed that tolfenamic acid dissolved most rapidly at the onset of in vitro lipolysis and its dissolution rate slowed continuously from then on, while titration data (Figure 2) showed that FFA production was also greatest at the onset of digestion and slowed continuously from then on. Comparing the quantity of dissolved tolfenamic acid during digestion directly with the quantity of liberated FFA reveals a linear relationship between the two. This effect has been observed previously, in the context of milk digestion in the presence of halofantrine as a weakly basic drug, in that case due to fatty acid ion-pair formation and the capacity of micellar structures to dissolve the ion-pair [8]. It should be reinforced that in the case of tolfenamic acid the effect is not due to lipophilic ion-pairing, but due to improved solubility in colloidal structures containing fatty acid and monoglyceride digestion products, and the more fatty acid and monoglyceride was generated, the greater the solubilisation capacity of those mixed micellar structures to dissolve molecular drug. The comparative influence of monoglyceride and the two mols of fatty acid produced upon digestion of the triglycerides is not clear from this study, however the monotonic increase in solubilisation with fatty acid content (measured by titration) indicates that both fatty acid and monoglyceride are probably contributing similarly to the magnitude of solubilisation, which makes sense for a non-ionisable drug. Additionally, dissolution of tolfenamic acid also stopped when the production of FFAs plateaued. This indicates that the dissolution of tolfenamic acid during the digestion of reconstituted infant formula was rate-limited by the production of FFAs and monoglycerides, not dissolution of the crystalline drug.

In addition to lipids, infant formula contains high levels of proteins, potassium, chloride, and other components. These components may influence drug solubilisation through interactions with the drug or the digestion process. However, it should be noted that in previous studies using casein that there was no impact of digestion on drug solubilisation [8] and we also note from the current study that the solubilisation of drug depended only on lipolysis, with much greater solubility achieved after lipolysis while most of the other components mentioned above remain constant. Consequently, while we agree that other components of infant formula have some potential to influence digestion and drug solubilisation, it is clear that in this case the behaviour is dominated by lipolysis.

It is worth noting that the dissolution of tolfenamic acid was evaluated without the inclusion of a gastric digestion phase. Although the solubility of tolfenamic acid in gastric phase in the presence of gastric enzymes has not been previously reported (at least to the authors’ knowledge), it is reasonable to expect that tolfenamic acid would exhibit poor solubility under gastric conditions. While the addition of gastric lipase may liberate some free fatty acids capable to inducing some degree of drug solubilisation, the extent of which lipids are digested by gastric lipase is typically less than 20–25%. Furthermore, it is unlikely that ionisation of tolfenamic acid is the primary factor governing its solubility in the small intestinal pH as the solubility in FaSSIF at pH 6.5 (0.063 mg/mL) [30] was <20% that of the solubility in undigested IF (0.34 mg/mL). Therefore, the dissolution of tolfenamic acid in IF is driven predominantly by the release of free fatty acids during digestion in the intestinal phase rather than pH-dependent drug ionisation. The particle size distribution of the reference drug material was not measured directly, but it was not micronised—the dissolution of co-administered solid drug particles is often considered a barrier to development of products or approaches such as the ‘Chasing Principle’ where solid drug is co-administered with a lipid formulation, but not co-formulated [9]. While this concept makes sense where drugs would normally be recommended to be taken with a meal to provide adequate drug solubilisation, the use of infant formula here in particular, and for an acidic drug, opens opportunities to explore formulations that would normally be ignored for acidic poorly water-soluble compounds although only one acidic drug has been described in the current work.

The findings therefore provide a novel experimental means to design formulations where the amount of fat required to dissolve the entire dose can be extrapolated from a single kinetic measurement or the end point of a series of measurements. In the case of the data in Figure 5, a linear regression of all of the data as a single dataset provides the equation that the %residual crystalline drug = −41.69 × [mmol fatty acid produced] + 102.79 and R2 correlation coefficient of 0.8497. Extrapolation of the plot of solubilised tolfenamic acid vs. titrated FFAs in Figure 5 indicates that 2.46 mmol of FFA would be required to completely solubilise a 25 mg dose of tolfenamic acid. Although three different fat-to-drug ratios were tested here, the use of a single profile of drug solubilisation at a single fat content, e.g., only 3.8% fat = 27.4 mg fat/mg drug (red dots in Figure 5) as a standard approach could be used effectively to estimate the total amount of fat required to dissolve the nominal dose of drug. It should be noted that this approach would likely yield different extrapolated amounts of FFA (and monoglyceride) required due to differences in affinity of different drugs for the micellar structures formed on digestion. Alternatively, the end points from titration of formulations to provide the amount of FFA produced, and separation of dissolved drug and quantification by HPLC, conducted at several ratios, would also provide points for extrapolation to the FFA axis to also indicate likely fat content necessary to dissolve the full dose of drug after digestion, without the need for in situ scattering measurements. We also note that analytically, in situ low-frequency Raman scattering has been shown previously to provide the same quantitative determination of drug solubilisation during digestion [10,31]. The broader applicability of this approach needs to be evaluated across a wider range of drugs in future.

## 5. Conclusions

This work showed that the solubility of tolfenamic acid in reconstituted infant formula was enhanced by digestion of the lipid components. This driving force for dissolution of crystalline drug during digestion manifested in dynamic solubilisation of tolfenamic acid during digestion when measured kinetically using either synchrotron SAXS measurements or separation of dissolved drug at select time points and HPLC. The digestion-driven solubilisation of tolfenamic acid was linked directly to the production of free fatty acids during in vitro digestion. The solubilising effect provided by free fatty acids cannot be attributed to ion-pairing interactions with tolfenamic acid and instead reflects the greater solubility of tolfenamic acid in long-chain lipid digestion products than in undigested long-chain lipids. This study reinforces the value of measuring the solubility of a poorly soluble drug in undigested and digested lipids when attempting to predict the solubilisation behaviour of the drug during gastrointestinal transit and provides a potential workflow to estimate the effective fat content required to completely dissolve a dose of drug into the digested formulation. Future efforts to more widely evaluate this framework both in vitro and in vivo will provide a generalised approach for co- co-formulation or co-administration of drugs with digestible lipid-based carriers such as infant formula.

## Data Availability

The original contributions presented in this study are included in the article/Supplementary Materials. Further inquiries can be directed to the corresponding author.

## Supplementary Materials

The following supporting information can be downloaded at: https://www.mdpi.com/article/10.3390/pharmaceutics18040480/s1, Table S1: Table of composition information for infant formula used in this study. Infant formula was reconstituted to three fat contents to provide different ratios of fat to tolfenamic acid (TA) in the samples. Figure S1: X-ray diffraction pattern of reference crystalline tolfenamic acid, with asterix indicating the major Bragg peak used for quantifying crystalline drug at *q* = 0.83 Å^−1^. Figure S2: X-ray diffraction patterns taken during digestion of TA in reconstituted infant formula showing the progres-sive decrease of diffraction intensity over time (best observed by the change in intensity of the peak at ~*q* = 0.4 Å^−1^ in this format) but no evidence of new diffraction peaks that would indicate a polymorphic transfor-mation during digestion. Arrows point to drug peaks while asterisks point to peaks from lamellar phase aris-ing from formation of calcium soaps during digestion.
