# Supplementary material for: Unexpected Effect of Digestion Products of Infant Formula in Enhancing the Solubilisation of Tolfenamic Acid During Digestion
Source: Pharmaceutics. 2026 Apr 14;18(4):480. doi: 10.3390/pharmaceutics18040480 (PMC13119033; doi:10.3390/pharmaceutics18040480)
Supplement: Supplementary file 1 [file pharmaceutics-18-00480-s001.zip › pharmaceutics-4233781-supplementary.pdf]

pharmaceutics MDPI

Unexpected effect of digestion products of infant formula in enhancing the solubilisation of tolfenamic acid during digestion

Thomas Eason, Malinda Salim, Vanessa Zann and Ben J. Boyd

Table S1 – Table of composition information for infant formula used in this study. Infant formula was reconstituted to three fat contents to provide different ratios of fat to tolfenamic acid (TA) in the samples.

**Nutritional information**	**Quantity per 100 mL of reconstituted infant formula**		
	**13.7 mg fat /mg TA**	**27.4 mg fat /mg TA**	**41.0 mg fat /mg TA**
Total fat	1.90 g	3.80 g	5.70 g
Protein	0.79 g	1.58 g	2.37 g
Carbohydrate	3.50 g	7.00 g	10.51 g
Galacto-oligosaccharide	0.24 g	0.47 g	0.71 g
Sodium	11.5 mg	23.0 mg	34.5 mg
Potassium	37.3 mg	74.6 mg	111.8 mg
Chloride	25.1 mg	50.1 mg	75.2 mg

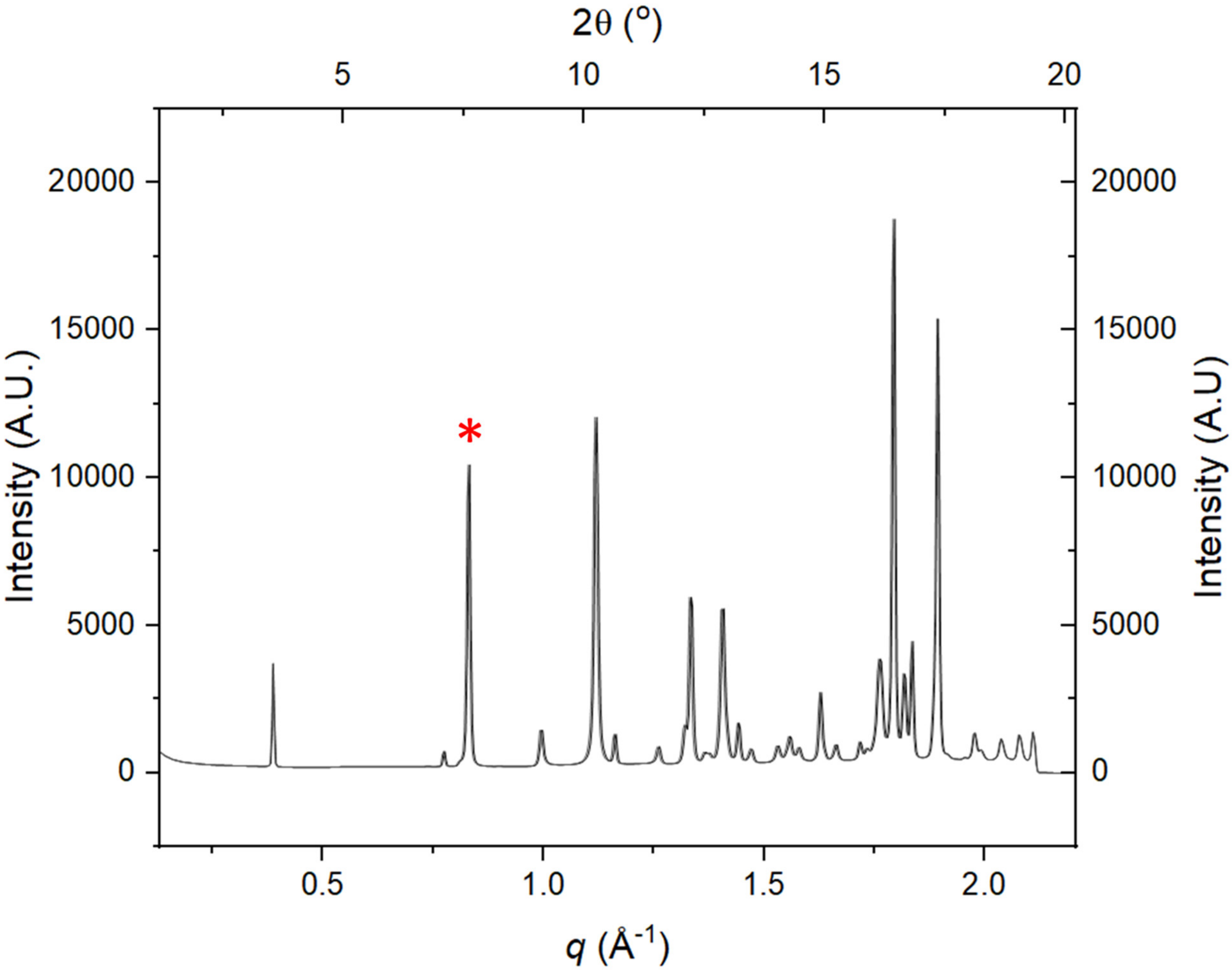

Figure S1 – X-ray diffraction pattern of reference crystalline tolfenamic acid, with asterix indicating the major Bragg peak used for quantifying crystalline drug at q = 0.83 $Å^{-1}$.

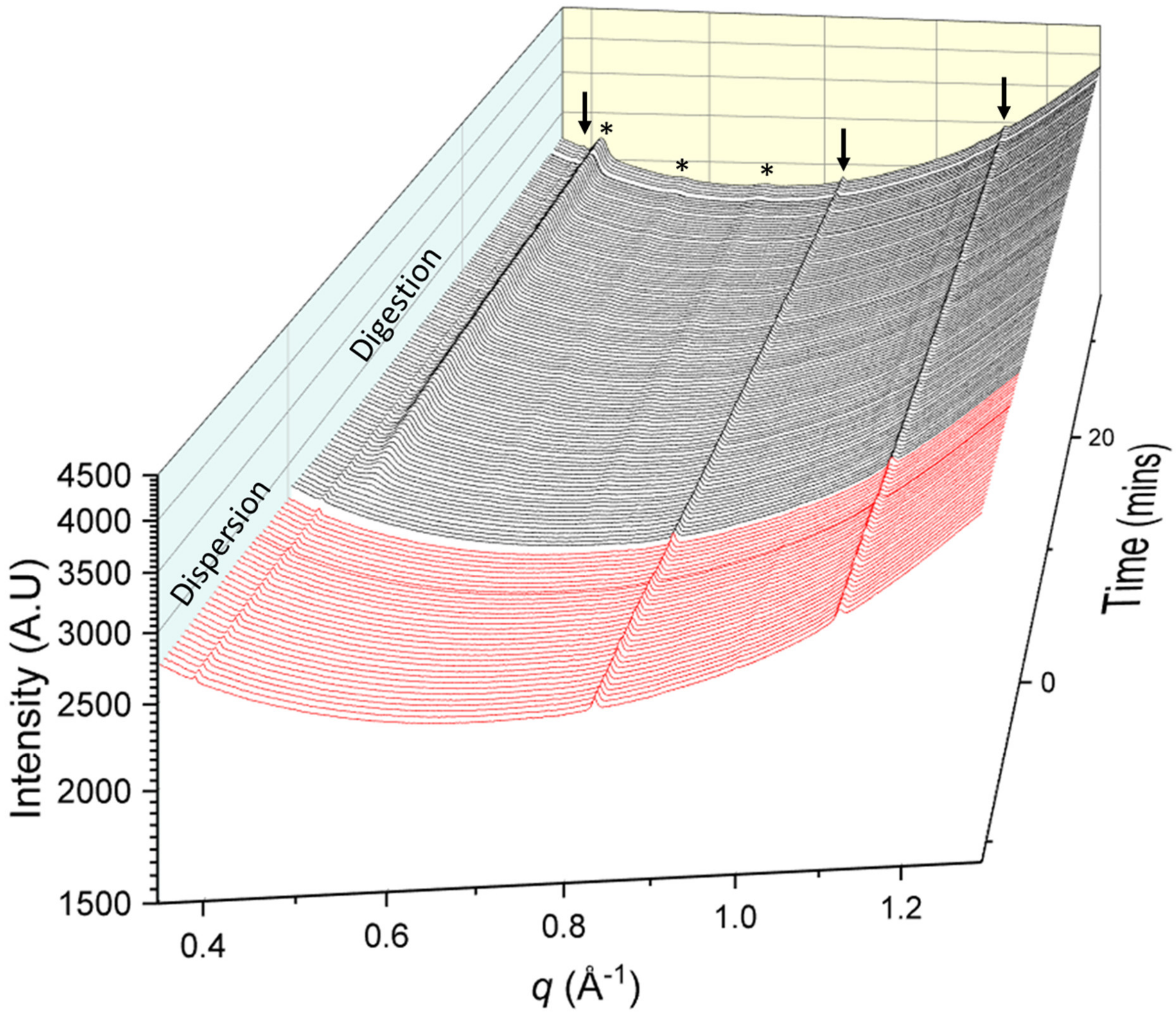

Figure S2 – X-ray diffraction patterns taken during digestion of TA in reconstituted infant formula showing the progressive decrease of diffraction intensity over time (best observed by the change in intensity of the peak at ~q = 0.4 $Å^{-1}$ in this format) but no evidence of new diffraction peaks that would indicate a polymorphic transformation during digestion. Arrows point to drug peaks while asterisks point to peaks from lamellar phase arising from formation of calcium soaps during digestion.
